# Les sarcomes des tissus mous: à propos de 33 cas

**DOI:** 10.11604/pamj.2015.22.374.8391

**Published:** 2015-12-16

**Authors:** Jiddou Abdou, Mustapha Elkabous, Hind M'rabti, Hassan Errihani

**Affiliations:** 1 Service d'Oncologie Médicale, Institut National d'Oncologie de Rabat, CHU Ibn Sina, BP 6213 Rabat, Maroc

**Keywords:** Sarcome des tissus mous, chimiothérapie, facteurs pronostiques, Soft tissue sarcoma, chimiotherapy, prognostic factors

## Abstract

L'objectif de cette étude est de rapporter les particularités épidémiologiques, cliniques, histologiques, thérapeutiques et évolutives des sarcomes des tissus mous à l'Institut National d'Oncologie et de définir les facteurs influençant la survie des patients. C'est une étude rétrospective de 33 cas de sarcome des tissus mous, colligés entre janvier 2008 et décembre 2010. Les critères d’éligibilité étaient un âge supérieur à 16 ans, une épreuve histologique d'un sarcome des tissus mous à l'exclusion des tumeurs stromales gastro-intestinales (GIST). Les items recueillis étaient: épidémiologiques, cliniques, histologiques, Radiologiques, et thérapeutiques. Des analyses univariées puis multivariées ont été réalisées à la recherche de facteurs influençant la survie à 2 ans. Il s'agit de 33 cas, 17 Hommes et 16 Femmes, l’âge moyen était de 43,21 ans (Extrêmes= 18-76 ans). La tumeur était localisée aux extrémités dans 24 cas (72,72%). Le type histologique prédominant était le Liposarcome dans 9 cas (27,27%). Le stade tumoral était localisé dans 25 cas (75,8%), métastatique dans 8 cas (24,2%). Vingt-cinq tumeurs ont été traitées chirurgicalement dont 21 cas (84%) de chirurgie conservatrice et 4 cas (16%) de chirurgie radicale. La radiothérapie a été réalisée chez 10 patients (30,3%). La chimiothérapie a été faite chez 20 patients. En analyse univariée les facteurs pronostiques étaient l’âge (p=0,03) et le stade tumoral (p=0,09). L’âge et le stade tumoral sont des facteurs pronostiques influençant la survie des sarcomes des tissus mous.

## Introduction

Les sarcomes des tissus mous (STM) sont des tumeurs rares qui présentent 1% des tumeurs solides de l'adulte, regroupent une grande variété de tumeurs malignes d'origine mésenchymateuse, survenant à tous les âges et dans des localisations multiples, leur prise en charge nécessite une approche multidisciplinaire dès le diagnostic initial, de préférence, dans des centres de référence [1]. Le traitement des STM Localisés est surtout chirurgical consistant en exérèse large avec une marge de 1 à 2 cm [1, 2]. La conformité de la procédure chirurgicale aux guides des bonnes pratiques cliniques est un facteur majeur et indépendant de prédiction de la survie sans progression des patients avec un STM et de la survie globale (SG) pour les patients atteints de liposarcomes [3]. L'objectif de cette étude est de rapporter les particularités épidémiologiques, cliniques, histologiques, thérapeutiques et évolutives des sarcomes des tissus mous à l'Institut National d'Oncologie et de définir les facteurs influençant la survie des patients.

## Méthodes

C'est une étude rétrospective de 33 cas de sarcome des tissus mous, colligés entre janvier 2008 et décembre 2010. Les critères d’éligibilité étaient un âge supérieur à 16 ans, une épreuve histologique d'un sarcome des tissus mous à l'exclusion des tumeurs stromales gastro-intestinales (GIST). Les items recueillis étaient: l'Age au moment du diagnostic, le sexe, la localisation tumorale et sa taille, le type histologique et son Grade, le stade tumoral, le traitement chirurgicale conservateur ou radical des formes localisées, la radiothérapie adjuvante ou palliative, la chimiothérapie (néoadjuvante ou palliative). Des analyses univariées puis multivariées ont été réalisées à la recherche de facteurs influençant la survie à 2 ans. Les médianes de survie sans récidive et de la survie globale ont été calculées selon la méthode de Kaplan Meier. Le test log-Rank a été utilisé pour la comparaison des courbes.

## Résultats

### Analyses descriptives (Tableau 1)

Il s'agit de 33 cas, 17Hommes et 16 Femmes, l’âge moyen était de 43,21 ans (Extrêmes= 18-76 ans), une tuméfaction était présente dans 32 (96,6%) cas, la taille tumorale moyenne était de 11 cm (extrêmes=5-18 cm), la tumeur était localisée aux extrémités dans 24 cas (72,72%) des cas, au niveau du tronc dans 6 cas (18,18%), 2 cas (6,07%) au niveau Rétropéritonéal et 1 cas (3,03%) au niveau de la tête et cou. Le bilan radiologique initial comportait une tomodensitométrie (TDM) du site tumoral dans 50% des cas, une Imagerie par résonnance magnétique (IRM) dans 9 cas (27,27%), une TDM thoracique dans 22 cas (66,7%); une biopsie initiale a été faite chez 17(51,51%) patients, les autres ont bénéficiés d'une biopsie exérèse. Les types histologiques prédominant étaient le liposarcome dans 9 cas (27,27%), le synovialosarcome dans 9 cas (27,27%), le Léiomyosarcome dans 8 cas (24,24%), les autres types histologiques 7 cas (18,18%) (Rhabdomyosarcome 2 cas, 1 PNET (Peripheral neuroectodermal tumor), 1 Fibrosarcome, 1 Dermato fibrosarcome (DMFS), 1 sarcome pléomorphe et 1 Sarcome indifférencié). La tumeur était localisée dans 25 cas (75,8%), métastatique dans 8 cas (24,2%), toutes les métastases étaient pulmonaires, seules dans 6 cas, associées à un autre site dans 2 cas (1 OS, 1 Surrénales). Tous les vingt-cinq cas localisés ont eu une chirurgie sur la tumeur primitive dont 21 (84%) cas de chirurgie conservatrice et 4 (16%) cas de chirurgie radicale. La limite de l'exérèse était > 1 cm dans un seul cas, et <1 cm pour les autres cas (24 cas, 96%). La Radiothérapie a été réalisée chez 10 patients (30,3%) dont 9 cas en postopératoire et 1 cas palliatif. La chimiothérapie a été réalisée chez 20 patients (60,6%) dont 3 cas en situation néoadjuvante et 17 cas en situation palliative. La chimiothérapie palliative était à base d'Antracycline dans 17 cas (85%) dont 8 cas (47,05%) en monothérapie et 9 cas (50,95%) en poly chimiothérapie; 7 patients (35%) ont eu une réponse objective à la chimiothérapie (2 réponses partielles, 5 Stabilités), 8 patients (40%) ont progressés après la chimiothérapie, la réponse à la chimiothérapie n'a pas été Précisée chez 5 patients. Parmi les 25 patients dont Les tumeurs étaient Localisées, 12 (48%) ont Rechuté après un délai moyen de 6 mois (Extrême=1-13 mois) au niveau local dans 2 cas (20%) et à distance dans 10 cas (80%). Le siège des Métastases était le poumon dans 9 cas, associé à l'os dans 1 cas et au foie dans 1 autre cas, 1 seul cas de rechute métastatique isolée au niveau surrénalien. Les rechutes locales ont été traitées par chirurgie avec radiothérapie dans 2 cas. Les rechutes métastatiques ont été traitées par la chimiothérapie. Le suivi médian était de 19,5 mois (Extrêmes=2-76 mois). La médiane de survie sans progression et de survie globale était 12 et 19 mois respectivement.

**Tableau 1:** Analyses descriptives des patients présentant un sarcome des tissus mous

Caractéristiques	Nombre (%)
Sarcomes des tissus mous	33 (100%)
Age > 40 ans	16 (48,48%)
Age ≤ 40 ans	17 (51,52%)
**Sexe**	
Hommes	17 (51,52%)
Femmes	16 ( 48,48%)
**Taille tumorale**	
> 5 cm	23 (69,69%)
≤ 5 cm	10 (30,31%)
**Localisations**	
Extrémités	24 (72,72%)
Tronc	6 (18,18%)
Rétropéritoine	2 (6,07%)
Tête et cou	1( 3,03%)
**Type histologiques**	
Liposarcome	9 (27,27%)
Synovialosarcome	9 (27,27%)
Léiomyosarcome	8 (24,25%)
Autres types	7 ( 21,21%)
**Grade histologique**	
Grade 1	5 (15,15%)
Grade 2	15 (45,45%)
Grade 3	13 (39,4%)
**Stade**	
Localisé	25 (75,75%)
Métastatique	8 ( 24,25%)
**Chirurgie:**	25 (75,75%)
Conservatrice	21 (84%)
Radicale	4 ( 16%)
**Radiothérapie**	10 (30,3%)
Adjuvante	9 (90%)
Palliative	1 ( 10%)
**Chimiothérapie**	20 (60,6%)
Néoadjuvante	3 (15%)
Palliative	17 (85%)

### Les Analyses univariées et multivariées (Tableau 2)

Nous avons utilisé une régression logistique simple pour rechercher les facteurs influençant la survie a 2ans: les facteurs ayant un p-value <0,30 en analyse univariée on étaient introduits dans l'analyse multivariée. On a retenu comme facteurs influençant la survie à 2ans, ceux qui ont un p-value <0,05 en analyse multivariée. En analyse univariée, les facteurs qui semblent influencer la survie à 2ans sont: l’âge (OR=0,19 IC95%: 0,04-0,85; p=0,03), Le stade de la maladie (OR=0,14 IC95%: 0,01-1,36; p=0,09) et la taille tumorale (OR=0,40 IC95%: 0,08-2,06; p=0,27). L'analyse multivariée a montré que seul l’âge est un facteur de mauvais pronostic (OR=0,15 IC95%: 0,03-0,82; p=0,28).

**Tableau 2:** Analyses univariées et multivariées

Analyses univariées				Analyses multivariées		
	0R	IC	P	OR	IC	P
Age jeune	0,19	0,04-0,85	0,03	0,15	0,03-0,82	0,028
Limites	0,82	0,O4-14.4	0,89			
Grade	0,77	0,11-5,34	0,79			
Stade	0,14	0,015-1,36	0,09	0,12	0,01-1,52	0,10
Taille	0,40	0,O8-2,06	0,27	0,60	0,09-3,93	0,06

En analyse univariée, les facteurs qui semblent influencer la survie à 2ans sont: l’âge (p =0,03), Le stade de la maladie (p=0,09) et la taille tumorale ( p=0,27).

L'analyse multivariée a montré que seul l’âge est un facteur de mauvais pronostic (p=0,28)

### Le Test Log-Rank

**Figure 1:**
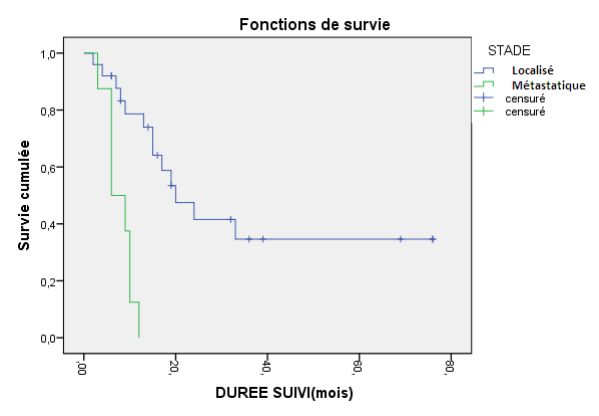
Courbe de survie en fonction du stade tumoral

Les résultats sont en concordance avec ceux obtenus par la méthode de régression logistique simple, le facteur pronostique était le stade tumoral, la médiane de survie était de 20 mois dans les stades localisés versus 10 mois dans les stades métastatiques (p=0,0001) ( Figure 1). Pour l’âge la différence n’était pas significative (p=0,06).

### Courbe de Survie (Figure 2)

Les médianes de survie sans récidive et de la survie globale ont été calculées selon la méthode de Kaplan Meier. Après un suivi moyen de 19,5 mois, la médiane de survie sans progression était de 12 mois et la médiane de survie globale était de 19 mois.

**Figure 2:**
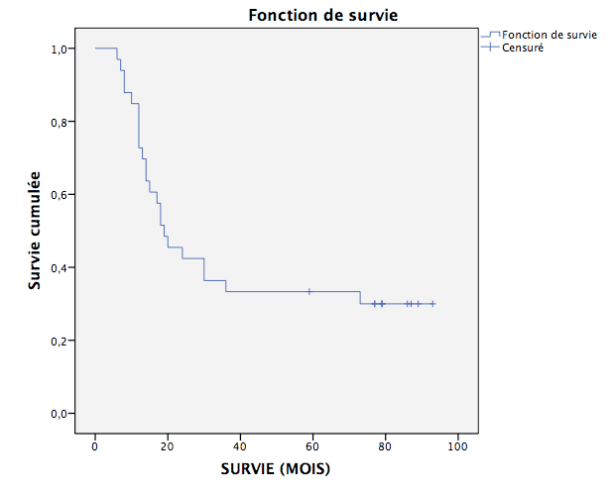
Courbe de survie globale

## Discussion

Les STM sont des tumeurs rares, durant la période de notre étude, 14100 cas de cancer, tout type confondu, ont été enregistrés dans notre institution dont 150 cas de sarcome des tissus mous ce qui représente 1% des cancers chez l'adulte comme décrit dans la littérature [1, 2], seul 33 cas ont eu un suivi et ont fait l'objectif de notre étude. L'incidence mondiale varie entre 3-5/100000 H par an [1, 2]. Ces tumeurs peuvent survenir à tout âge mais le plus souvent après 40 ans, le moyen d’âge dans notre étude était 43,21 ans. Il existe une légère prédominance masculine dans notre étude (sexe ratio 1,06 Homme pour 1 Femme). Le facteur causal des STM reste inconnu, cependant certaines maladies génétiques et des facteurs environnementaux sont reconnus comme facteurs prédisposant. La radiothérapie surtout à haute dose est un facteur bien connu de survenu de sarcome des tissus mous [1, 4]. Dans notre étude aucune enquête étiologique n'a été faite, mais on n'a pas eu de notion d'antécédents personnels ni familiaux de maladie génétique. Aucun de nos patients n'avait d'antécédent de radiothérapie. Les localisations les plus fréquentes sont les membres, le tronc, le rétropéritoine puis la tête et le cou [1, 4]. Dans notre étude les extrémités représentaient plus de deux tiers avec 24 cas (72,72%), le tronc 6 cas (18,18%), le Rétropéritoine 2 cas (6,07%), et la tête et cou 1 cas (3,03%) ce qui est décrit dans la littérature. Les Manifestations cliniques révélatrices des sarcomes sont les douleurs et les tuméfactions, dans notre étude la taille tumorale variait entre 5 et 18 cm (moyenne =11 cm).

L'hétérogénéité des sarcomes des tissus mous rend compte de la complexité de l’étude histologique qui doit être faite par un pathologiste spécialisé dans ce domaine si non un deuxième avis doit être demandé systématiquement pour s'assurer du diagnostic [2]. Dans notre étude le type histologique le plus fréquent était le liposarcome dans 9 cas (27,27%), le synovialosarcome 9 cas (27,27%), le léiomyosarcome 8 cas (24,25%), et 7 cas (21,21%) autres types histologiques. Le diagnostic histologique de tous nos patients a été posé dans d'autres structures et dans 1 seul cas une relecture a été faite objectivant une discordance partielle. La prise en charge des STM doit être multidisciplinaire dès l’étape initiale du diagnostic avec la réalisation d'un bilan radiologique avant la biopsie. L'IRM est l'examen de référence car elle permet l’étude des rapports anatomiques avec les structures adjacentes, l'extension locorégionale, de guider la biopsie et d’évaluer la résécabilité de la tumeur. Dans notre étude cet examen a été réalisé seulement chez 9 patients (27,27%), ceci peut être expliqué par le problème de disponibilité et du coût de cet examen. Suite au bilan radiologique la biopsie s'impose pour la réalisation de l’étude histologique, cependant des petites lésions superficielles peuvent bénéficier d'une biopsie exérèse d'emblée en évitant l'effraction tumorale [4]. Seulement 17 (51,51%) de nos patients ont bénéficié d'une biopsie initiale, une exérèse d'emblée a été faite dans les autres cas (48,49%).

Le traitement standard des sarcomes des tissus mous localisés résécables est la chirurgie qui consiste en une exérèse large en un bloc emportant la cicatrice de biopsie et la tumeur avec une marge circonférentielle de tissu non tumoral de 2 cm ou une barrière anatomique (résection R0). La qualité de la chirurgie est déterminée par l'examen histologique des marges de la pièce opératoire, si les marges sont envahies, une reprise chirurgicale doit être faite [1, 2, 4].

Les marges chirurgicales insuffisantes sont les facteurs de récidive locale [1, 4]; dans notre étude seul un cas (4%) a bénéficié d'une exérèse avec marge saine supérieure à 1 cm, pour les vingt et quatre autres patients avec sarcome localisé ayant bénéficié d'un traitement conservateur, les marges étaient envahies, une radiothérapie adjuvante a été réalisée chez 10 (40%) d′entre eux. Une étude randomisée a démontré l’équivalence en termes de survie sans récidive et de survie globale de la résection non radicale associée à la radiothérapie et l'amputation dans les sarcomes localisés des membres [1]. Deux études randomisées ont démontré le bénéfice de la radiothérapie adjuvante en termes de survie sans récidive mais pas en termes de survie globale [1]. Cette radiothérapie adjuvante doit être réservée pour les STM de plus de 5 cm, profonds ou de haut grade (G2, 3). Les lésions de 5 cm ou moins, superficielles et de bas grade ne nécessiteront pas de radiothérapie après une exérèse chirurgicale en marges saines [2, 4].

Aucun de nos patients n'a reçu une chimiothérapie adjuvante car elle n'est pas un standard. Une méta analyse de données de 1568 patients dans 14 essais et un essai italien ont démontré un bénéfice d'une chimiothérapie adjuvante [5], cependant deux essais de phase 3 randomisés de l'EORTC (Eurpean Organisation for Research and Treatment of Cancer) évaluant la chimiothérapie adjuvante n'ont pas démontré son bénéfice en termes de survie globale, mais l’ analyse de sous-groupes a démonté que Les patients de sexe masculin ainsi que les patients dont l’âge > 40 ans ont un bénéfice en termes de survie sans progression, alors que le sexe féminin et l’âge < 40 ans sont associés à une survie globale médiocre; les patients avec une résection R1 ont une meilleure survie sans progression et survie globale après la chimio adjuvante [6]. Dans les STM localement avancés le but de la chimiothérapie néoadjuvante est de diminuer la taille tumorale pour permettre un traitement conservateur et de tester la sensibilité tumorale à cette chimiothérapie. Dans notre étude 3 patients (12%) ont bénéficiés d'une chimiothérapie néoadjuvante par Doxorubicine à la dose de 75 mg/m^2^ toutes les 3 semaines avec une réponse partielle dans un cas et une stabilité dans les 2 autres cas. Une chimiothérapie à base d'antracycline avec un schéma dose dense, donne une réponse objective à 94% avec un taux de résection R0 à 82% et un taux de survie sans progression et de survie globale à 5 ans de 48% et 64% respectivement [7]. Le bénéfice de la chimiothérapie néoadjuvante est augmenté par l'hyperthermie locale [4]. Dans notre étude, 17 patients (51,51%) ont reçu une chimiothérapie palliative dont 8 patients (47,05%) étaient métastatiques d'emblée et 9 patients (52,95%) étaient en rechute métastatique. La chimiothérapie était à base d'antracycline dans 15 cas (88,23%). Une monochimiothérapie a été réalisée chez 10 patients (58,82%) et une Polychimiothérapie chez 7 patients (47,18%).

Le traitement des STM métastatiques est la chimiothérapie palliative; aux 3 substances classiquement actives dans les STM qui sont la Doxorubicine, l'Ifosfamide et la Dacarbazine s'ajoute une nouvelle molécule, la Trabectédine. La Doxorubicine est l'agent le plus utilisé en première ligne à des doses entre 60 et 75 mg/m^2^toutes les 3 semaines avec un taux d'activité entre 16 et 30% [4, 8], sa cardiotoxicité cumulative en limite l'usage à long terme et pour pallier à cette cardiotoxicité d'autres formulations ont été développées dont la Doxorubicine pegylée qui a démontré, à la dose de 50 mg/m^2^ toutes les 4 semaines, une efficacité équivalente à la Doxorubicine avec une toxicité moindre et qui est surtout cutanéomuqueuse à type syndrome main pied [4]. Une nouvelle formulation intéressante est l'Aldoxorubicine, Une pro drogue de la Doxorubicine dérivée de sa liaison avec un lien sensible au milieu acide, après le passage en circulation sanguine il se lie rapidement et de façon covalente à l'albumine et passe préférentiellement en intra tumoral dont le milieu acide provoque le clivage du lien et la Doxorubicine est ainsi délivrée directement au niveau tumoral ce qui permet de contourner le cœur et éviter la toxicité cardiaque. Dans une étude de phase I l'Aldoxorubicine à la dose de 350mg/m^2^toutes les 3 semaines, a démontré un taux de réponse objective de 76% dans les sarcomes des tissus mous. Récemment une étude de phase II randomisée a comparé en première ligne la Doxorubicine (75 mg/m^2^ j1, 6 cycles) et l'Aldoxorubicine(350 mg/m^2^ J1 toutes les 3 semaines) jusqu’à progression. Cent vingt-trois patients ont été inclus. Un taux de réponse objective de 25% versus 0% et une survie sans progression (PFS) médiane de 5,6 mois versus 2,8 mois (p=.02) sont observés en faveur de l'Aldoxorubicine. L'Aldoxorubicine entraîne cependant plus d’épisodes de neutropénie de grade 3-4 (29% vs 15%), mais moins de neutropénie fébrile grade 3-4 (14% vs 18%), pas de cardiotoxicité aigue dans les deux bras mais une diminution de la fraction d’éjection du ventricule gauche (FEVG) inférieure à 50% chez 3 patients parmi les 40 qui ont reçus la Doxorubicine [9]. Une étude de phase III est en cours. L'Ifosfamide est un agent alkylant de la famille des oxazaphosphorines qui a une activité dose dépendante dans les STM. Les fortes doses de 6 à 14 g/m^2^ donnent des taux de réponse croissant avec la dose administrée mais nécessitant l'utilisation des facteurs de croissance granulocytaires, son efficacité est aussi fonction de son mode d'administration, en effet le mode bolus est plus actif que l'administration en continue [4]. Les taux de réponse sont de l'ordre de 38% pour des doses de 5 à 8g /m^2^ toutes les 3 semaines [8]. Sa toxicité est essentiellement hématologique, vésicale, rénale, hépatique et neurologique.

La Trabectédine, Extrait d'un tunicier de mère (l'ascidie Ecteinascidia turbinata) et actuellement obtenue par synthèse, est un agent du petit sillon de l'ADN empêchant la progression du cycle cellulaire, son activité a été démontrée initialement chez les patients atteints de STM en échappement aux antracyclines avec une réponse objective de 38%, puis confirmée par des études rétrospectives et prospectives, la réponse était médiocre de l'ordre de 4 à 8% mais avec une stabilité prolongée chez plus de 20% des patients [8], actuellement a démontré, dans une études de phase II randomisée, son efficacité en première ligne dans le léiomyosarcome utérin en association avec la Doxorubicine avec une réponse objective de 87% [10]. La Dacarbazine donne des réponses objectives de 18% à la dose de 1200 mg/m^2^, la toxicité hématologique est plus sévère en administration en bolus qu'en administration fractionnée [8]. Le standard est de faire une mono chimiothérapie par antracycline en 1 ère ligne dans les sarcomes des tissus mous, les associations de Polychimiothérapie à base d'antracycline donnent des réponses objectives entre 19 et 34% et n'ont pas démontré de bénéfice en survie globale par rapport à la monothérapie à base de Doxorubicine, Ceci a été confirmé par une métanalyse ainsi qu'un essai randomisé de phase III de l'EORTC [11], Cependant, une nouvelle association, semble intéressante, de la Doxorubicine avec l'Evofosfamide (TH 302) qui est un agent alkylant proche de l'Ifosfamide, actif seulement en milieu d'hypoxie. C'est une prodrogue de la moutarde Bromo-isophospharamide (Br-ISPM). Le TH 302 est réduit à son site nitroimidazole par des réductases intracellulaires dans des conditions hypoxiques provoquant ainsi la libération de la Br-ISPM qui est le métabolite actif et qui agit par la formation de ponts stables, par des liaisons covalentes, entre les deux chaines de l'ADN empêchant ainsi son dédoublement et par conséquence empêche la mitose. Dans une étude de Phase 2 la Doxorubicine à la dose de 75mg /m^2^toutes les 3 semaines était associée au TH 302 à la dose de 300 mg/m^2^ J1 et J8 tous les 21 jours chez 92 patients présentant des STM métastatiques naïfs de toute chimiothérapie, après 6 cycles, les patients répondeurs ou stables recevaient le TH 302 en maintenance. Le taux de réponse objective était de 36%, le taux de survie sans progression à 6 mois était de 58%, les médianes de survie sans progression et de survie globale étaient de 6,5 mois et de 21 mois respectivement. Au cours de la phase de maintenance la réponse objective a été améliorée de 12,5%. Les principales toxicités étaient la fatigue, les nausées, la toxicité cutanéomuqueuse, l'anémie, la leucopénie et la thrombopénie mais moins sévères en phase de maintenance. Il n'y avait pas de toxicité cardiaque, hépatique ni rénale liée au TH 302, une phase III est en cours [12]. La Polychimiothérapie reste préférable pour les patients avec des métastases potentiellement résécables.

Le taux de réponse objective de la chimiothérapie de 1^ère^ ligne dans les sarcomes des tissus mous métastatiques varie entre 15 et 34% [11]. Dans notre étude, 4 patients (23%) parmi les 17 ayant reçu la chimiothérapie palliative, ont eu une réponse à la chimiothérapie (2 réponses partielles et 2 stabilités), 8 patients (47,05%) ont progressé, la réponse n'a pas été déterminée chez 5 patients (29,4%). Quatre patients (23%) ont développé des toxicités hématologiques de grade 3-4 avec un seul cas (5%) de neutropénie fébrile. Cinq patients en progression (62,5%) ont eu une chimiothérapie de deuxième ligne par Ifosfamide dans 2 cas, Ifosfamide + Etoposide dans un cas, Doxorubicine + Ifosfamide dans un cas, et par Dacarbazine dans un cas. Actuellement le traitement de deuxième ligne des STM après échappement aux antracyclines est fonction du type histologique et de l'existence d'une anomalie moléculaire potentiellement sensible à une thérapie ciblée. La Trabectédine a démontré, dans des études de phase II, une activité en deuxième ligne dans plusieurs types histologiques comme le léiomyosarcome utérin [13], le liposarcome myxoïde [13, 14], et les Sarcomes à translocations [15]. Les associations de Gemcitabine avec le Docétaxel ou la Dacarbazine sont efficaces en deuxième ligne dans le léiomyosarcome utérin après échappement aux antracyclines [8]. Le Pazopanib, un inhibiteur multikinase bloquant la transduction des messages transmis par les récepteurs du VEGF et du PDGF ainsi que les voies du KIT et du FLT3 et bloquant ainsi l'angiogenèse tumorale, représente une nouvelle option thérapeutique en deuxième ligne des sarcomes des tissus mous à l'exception du liposarcome. Dans une étude de Phase III, le Pazopanib à la posologie de 800 mg/j a été comparé avec placebo chez des patients présentant des sarcomes des tissus mous en progression après une chimiothérapie standard. Trois cent soixante-neuf patients ont été randomisés, l'objectif primaire était la survie sans progression. La médiane de survie sans progression était de 4,6 mois pour le Pazopanib contre 1,6 mois dans le bras placebo (p < 0001) avec plus de toxicités dans le bras de Pazopanib (Fatigue, nausées, diarrhées et l'HTA), le taux de réponse objective était de 73% pour le Pazopanib. C'est la seule étude de phase III qui a démontré une amélioration de la survie sans progression dans les STM [16].

Pour les patients ayant obtenu des réponses partielles ou une stabilisation après la chimiothérapie de 1ere ligne, le concept d'un traitement de maintenance a été exploré par un essai de Phase III évaluant le Ridaforolimus, un inhibiteur de la voie m-TOR (mammalian Target of Rapamycin), en maintenance chez les patients répondeurs après une chimiothérapie dans les sarcomes des tissus mous. Cet essai était positif démontrant une amélioration significative de la survie sans progression, l'objectif primaire de l'essai [17]. Les sarcomes des tissus mous métastatiques sont de mauvais pronostic avec des taux de survie sans progression à 6 mois variant entre 30 et 56% selon le type histologique et les substances utilisées [18], la survie globale est autour de 12 mois [19]. Dans notre série, après un suivi médian de 19,5 mois (Extrêmes=2-76 mois), La médiane de survie sans progression et de survie globale était 12 et 19 mois respectivement. Les facteurs influençant la survie étaient le stade tumoral (p < 0,0001) et l’âge (p=0,067).

## Conclusion

Les sarcomes des tissus mous sont des tumeurs rares, leur prise en charge nécessite une approche multidisciplinaire dès l’étape initiale du diagnostic, l’âge et le stade tumoral sont des facteurs pronostiques influençant la survie des patients. Notre étude est rétrospective et de petit effectif mais nos résultats sont concordant avec ceux décrits dans la littérature.
